# Dynamic Modulation of Coupled Plasmon Resonances in Antimony-Doped Tin Oxide Nanorod Metamaterial by Charge Carrier Injection

**DOI:** 10.1021/acs.nanolett.5c01485

**Published:** 2025-05-19

**Authors:** Thomas Herzog, Atefeh Habibpourmoghadam, Nele Pannewitz, Yaşar Krysiak, Irene Morales, Sonja Locmelis, Antonio Calà Lesina, Sebastian Polarz

**Affiliations:** † Institute of Inorganic Chemistry, 26555 Leibniz University Hannover, Callinstraße 3-9, 30167 Hannover, Germany; ‡ Cluster of Excellence PhoenixD, Leibniz University Hannover, Welfengarten 1A, 30167 Hannover, Germany; § Hannover Centre for Optical Technologies, Leibniz University Hannover, Hannover 30167, Germany; ∥ Institute for Transport and Automation Technology, Leibniz University Hannover, Garbsen 30823, Germany

**Keywords:** nanorod arrays, tunable plasmon resonance, tin oxide, metamaterial, cavity resonance mods, electro-optical modulation

## Abstract

Coupled plasmon resonances of adjacent particles in densely packed nanorod metamaterials can introduce extraordinary optical features, like cavity resonance modes. These modes, being commonly realized in metallic metamaterials, can be exploited for plasmonic sensing or optical modulation, due to strong optical and electrical field enhancement in the cavities. However, modulation of plasmon resonances in metallic nanostructures is limited due to their intrinsically high charge carrier concentration. We introduce a new metamaterial based on metal oxides, respectively an array composed of doped tin oxide nanorods featuring cavity resonance modes. By means of numerical simulations, the optical response of the fabricated plasmonic metamaterial is calculated and compared with the experimental findings in order to understand and clarify the nature of the optical modes. Moreover, dynamic modulation of the optical response is demonstrated by the electrochemical injection of electrons into the nanorods, thus paving the way to electro-optical modulation of such metamaterials.

## Graphical abstract

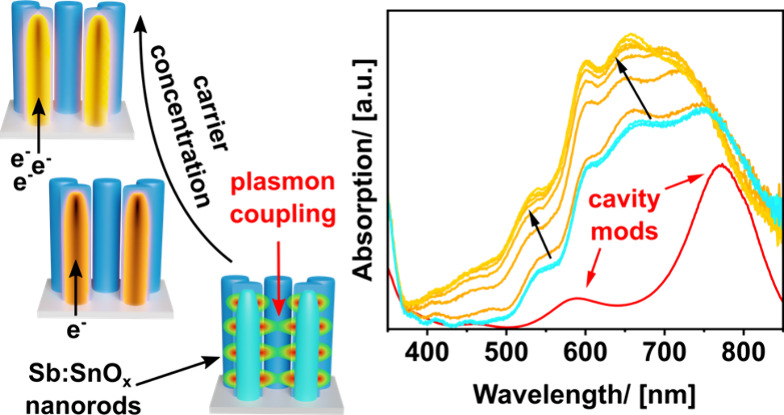

Plasmonic nanorod (or nanowire) metamaterials have gained significant relevance for emerging technologies, such as subwavelength imaging,^1,2^ sensing,^3,4^ spontaneous emission engineering,^53^ and light polarization control.^54^ This is due to their extraordinary light manipulation properties, including negative refraction,^5,6^ anisotropy, and hyperbolic optical dispersion.^47,55^ These artificial materials are constructed from periodic arrays of metal nanostructures with subwavelength spacing and height of the order of the wavelength of visible light.^7,8^ In general, the individual units in the metamaterials (UMs) exhibit localized surface plasmon resonances (LSPR), whose frequency and absorption range strongly depend on the material properties, size, shape, and surrounding environment.^9–13^ The arrangement of such nanoresonators in a regular pattern leads to collective behaviors under optical irradiation, and the emergence of extraordinary optical properties due to the interaction of the induced electric dipoles.^9,14,15^ In the simplest case of two adjacent UMs (spacing <50 nm), the coupling of spatially and spectrally overlapping dipolar resonances can lead to a shift and splitting of the resonance feature.^14^ When a larger number of UMs are in proximity (e.g., heptamers), a transition from isolated to collective plasmonic modes is observed.^1,4,16–22^ In nanorod array metamaterials, the coupling of transversal LSPR results in cavity resonant modes with electric fields concentrated between the nanorods, observable as absorption peaks in the optical spectrum.^23^ These modes are akin to cavity modes in metal–insulator–metal (MIM) waveguides.^24^ Cavity resonant modes form due to plasmons traveling along the gap between adjacent nanorods, which reflect from the underlying material and are characterized by multiple electric field hot spots in the longitudinal direction. In summary, when closely packed, nanorod arrays can exhibit cavity-type collective modes, where their mode number, spectral positions, and amplitudes are highly dependent on the nanorod geometry, spacing, and surrounding conditions.^23,25^

Doped metal oxide UMs commonly exhibit plasmon resonances in the near-infrared (NIR) spectral region.^13^ The plasmon resonance frequencies in doped metal oxides are not fixed at a specific wavelength but depend on the material’s composition and doping level.^12,13,26–30^ Moreover, metal oxide-based plasmonic materials allow for postsynthetic modulation of their plasmonic features based on reversibly altering their charge carrier concentration. In the search for new smart materials, this dynamic modulation can enable reversible manipulation of a metamaterial’s optical response, as changing a single nanorod’s plasmonic resonance can strongly influence the collective response of the metamaterial.^31–33^ Thus, enabling new switchable optical components for beam steering^31^ or phase and amplitude modulation.^34^ The modulation can be realized by injection of free charge carriers directly into the plasmonic UMs, either by utilizing a chemical reducing agent^35^ or by electrochemical doping.^36,37^

Here, we present a new tunable metamaterial based on highly doped metal oxide nanorod arrays. The small spacing between the nanorods (<10 nm) results in coupling of the transversal modes of adjacent nanorods and induces several higher-order cavity modes in the visible to NIR spectral range. The nature of the cavity modes and a strong field enhancement in the cavities between the nanorods are confirmed by numerical simulations. Through postsynthetic reversible electrochemical doping, the position of the cavity modes can be dynamically tuned, paving the way to electro-optically addressable metasurfaces.

The metal oxide nanorod arrays are synthesized by a hard-templating approach introduced by Martin et al.^38^ A porous anodic aluminum oxide (AAO) template is prepared on a coated silicon wafer, and the pores are filled with metal nanorods by an electrodeposition approach (see the Supporting Information).^39^ Antimony (Sb)-doped tin (Sn) oxide nanorods are obtained by controlled thermal oxidation of the Sn–Sb alloy nanorods. The Sb content is set to 10 atom % to achieve high-level n-type doping,^40^ inducing plasmon resonances in the NIR.^41,42^ Thermal oxidation is conducted with the nanorods still in the template to maintain their shape and arrangement (Figure 1a–d, Figure S1). Two different approaches are investigated. In the first approach, the nanorods are entirely confined in the AAO template, and oxygen can only access the nanorods from their tips (Figure 1a). In the second approach, the template surrounding the nanorods is slightly etched to open paths for oxygen diffusion to the lower nanorod sections (Figure 1b). For both oxidation processes, the so-called Kirkendall effect is noticeable.^43^ In the first approach (Figure 1a), the tips of the nanorods get oxidized at the beginning of the thermal oxidation, and an oxide layer is generated, acting as a diffusion barrier for ambient oxygen. Consequently, the mass flow of metallic Sn and Sb through the barrier oxide is faster than oxygen diffusion, and Sn/Sb are transported from the bottom part of the nanorods to their top, leaving voids in their lower parts (Figure 1c, orange arrows).

**Figure 1:**
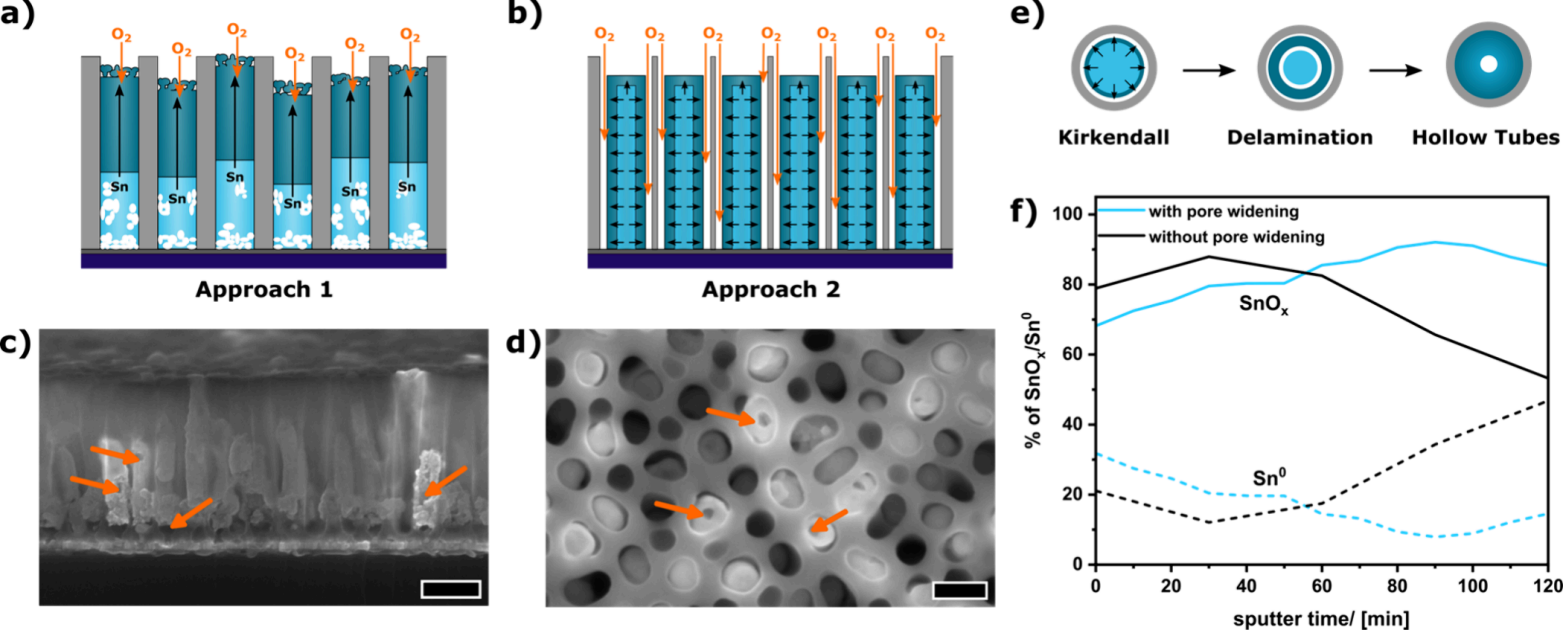
Schematic illustration of the Sn–Sb alloy nanorod oxidation with the nanorods fully encapsulated by the template (a, approach 1) and with oxygen diffusion channels (b, approach 2). Side-view scanning electron microscopy (SEM) image after the oxidation according to approach 1, showing the void formation (orange arrows) along the nanorods (c, scale bar 200 nm). Top-view SEM images of the nanorods of approach 2 (d, scale bar 100 nm), indicating the formation of some hollow nanorods (orange arrows), and the suggested formation mechanism (e). Relation of oxidized Sn (SnO_*x*_, solid lines) to metallic Sn^0^ (dotted line) along the nanorod long axis, determined by X-ray photoelectron spectroscopy (XPS) for approach 1 (black) and approach 2 (blue) (f).

In the second approach, due to the partial etching of the template, the nanorods are accessible for the ambient oxygen from the sides (Figure 1b), leading to a uniform oxide shell growth around the nanorods. Therefore, the Kirkendall effect and mass transport mainly occur from the nanorods’ interior to the outer shell.^44^ This results in the formation of some hollow nanorods (Figure 1d, orange arrows) due to void formation in their inner (Figure 1e), and breakage at the lower parts of the nanorods is inhibited (Figure S2). Investigation of the oxidation state of Sn along the nanorod length axis by X-ray photoelectron spectroscopy (XPS, Figure S3) indicates a strong oxidation gradient along the nanorods prepared by approach 1 (Figure 1f). This strong oxidation gradient may be useful for electrical applications (Figure S4).^39^ Utilizing approach 2, no oxidation gradient along the nanorods and less residual Sn^0^ is observed (Figure 1f).

After the oxidation, following approach 2 and acidic etching of the remaining template, defined and free-standing nanorods are obtained (Figure 2a). The nanorods are oriented side by side on the substrate and are arranged in a distorted hexagonal short-range order (Figure 2b). The spacing between adjacent nanorods is in the range of 5 – 10 nm, and they exhibit an average diameter of about 100 nm (Figure S5) and a length of around 660 nm. Some of the nanorods exhibit cracked tops, which indicate a core–shell-like structure of the nanorods, and also some hollow structures are visible (Figure 2b). EDX mapping of the constituent materials (Sn, Sb, and O) indicates a uniform distribution of the elements within the nanorods (Figure 2c). X-ray diffraction patterns of the nanorod array on the substrate (Figure 2d) show that the characteristic reflexes for the Sn (200) and Sn (101) - plane vanish after the oxidation process, and reflexes for the tetragonal SnO and the rutile SnO_2_ phase appear (Figure 2d). A wide range XRD pattern assigning all reflexes is shown in Figure S6 (Supporting Information). XPS Sn 3d core level spectra reveal that in the outer shell Sn in the oxidation state +IV is the dominant species, whereas in the interior Sn^2+^ is the dominant species with some traces of residual metallic Sn^0^ (Figure 2e, Figure S7).

**Figure 2:**
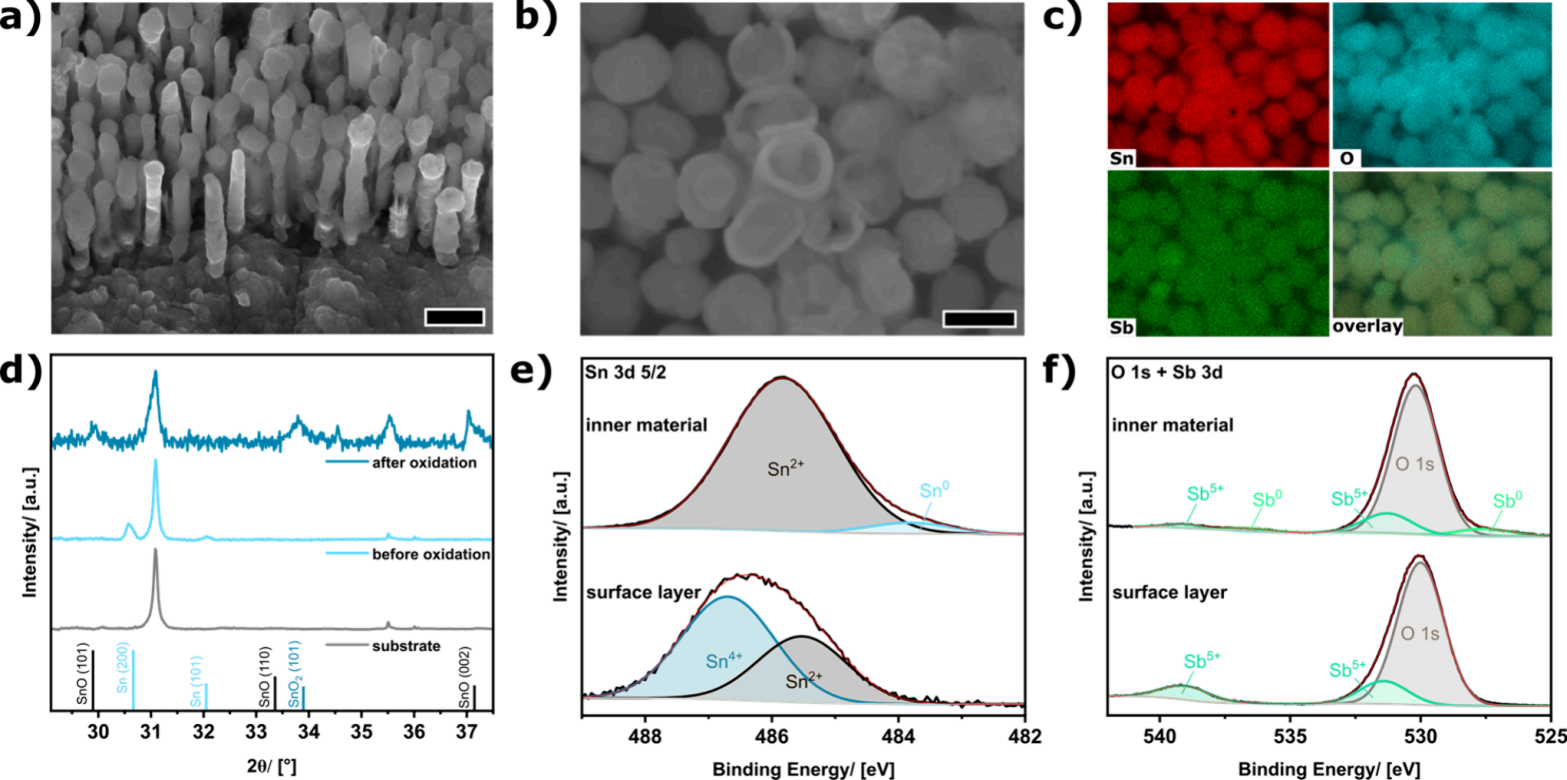
Tilted-view SEM image of the free-standing nanorod array after removal of the aluminum oxide template (a, scale bar 200 nm). Top-view SEM image of the nanorod array (b, scale bar 100 nm) with corresponding EDX mapping (c) of the constituent materials Sn (red), oxygen (blue) and Sb (green). X-ray diffraction (XRD) patterns (d) of the substrate (gray), the metallic nanorod array (light blue) and after oxidation of the nanorod array (dark blue) accompanied by reflex positions of β-Sn (ICSD 106072), SnO (ICSD 15516) and SnO_2_ (ICSD 39174). XPS Sn 3d (e), O 1s and Sb 3d (f) core level spectra of the surface layer and inner material of the oxidized nanorod array.

Deconvolution of the Sn spectra revealed that in the outer material, around 62% of the Sn is in the oxidation state + IV and about 38% is in the oxidation state +II. Since no reflexes for the Sn_3_O_4_ phase are found in the XRD pattern, the XPS results indicate that the outer layer is made from separated SnO and SnO_2_ grains. Deconvolution of the Sn spectrum of the inner material reveals that the core is made of SnO doped with about 5% of metallic Sn. According to the XPS measurements of the Sb 3d core level the dopant is present only in the oxidation state +V with some residuals of metallic Sb^0^ in the inner material (Figure 2f). In summary, the results reveal a core–shell structure of the nanorods (Figure 3a). The surface layer is composed of Sb-doped Sn^4+^ oxide (SnO_*x*_; *x* ≈ 2), whereas the inner material mainly comprises Sb-doped Sn^2+^ oxide (SnO_*x*_; *x* ≈ 1). The XPS additionally revealed that no Cr from the etching solution is incorporated in the nanorods (Figure S8).

**Figure 3:**
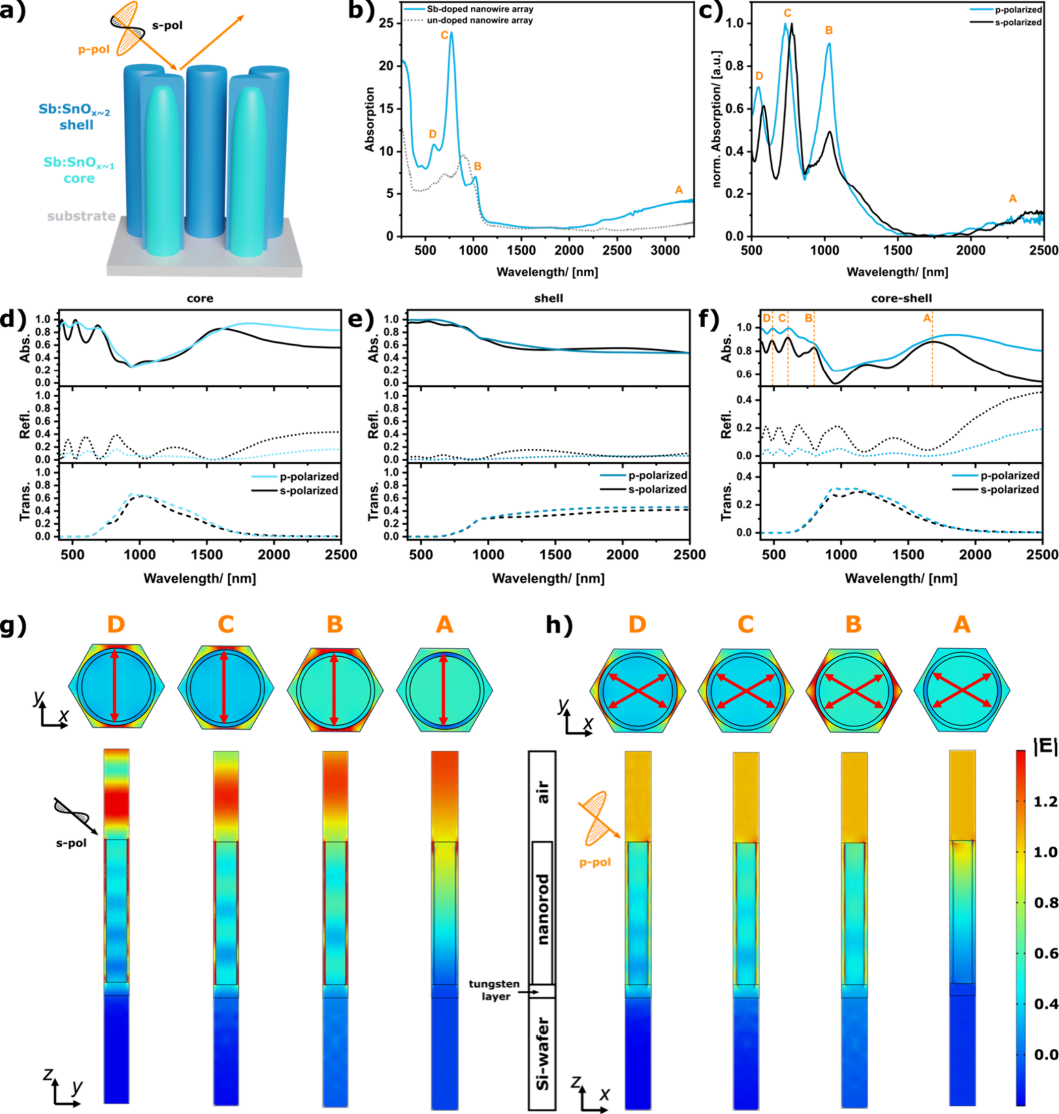
Schematic drawing of the nanorod array and measurement setup (a). Optical absorption spectra measured for unpolarized irradiance (b) of the nanorod array doped with Sb (blue) and of an undoped nanorod array (dotted gray). Optical absorption spectra of the Sb-doped nanorod array for s- (black) and p-polarized (blue) light (c). All absorption spectra are measured at an oblique angle of 45° in reflectance geometry. Simulated transmittance, reflectance and absorptance spectra of the nanorod array for the nanorod cores (d), for the nanorod shells (e) and for the core–shell structure (f). Simulated electric field enhancement in the nanorod metamaterial for s-polarized (g) and p-polarized (h) irradiation (amplitude of the incident electric field is assumed 1 V/m), indicating formation of cavity resonant modes .

This core–shell structure, in combination with the arrangement of the nanorods, results in several different plasmonic modes in the absorption spectrum (Figure 3b, blue). The effect of the Sb-doping is apparent, when comparing the NIR range of the spectra. In comparison with the undoped sample (Figure 3b, dotted gray), a highlighted plasmonic feature with a maximum around 3200 nm is found for the doped array (**A**). A plasmonic resonance feature in the NIR range is typical for highly doped metal oxides.^10,35,45,46^ Absorption peaks ranging from 500 to 1100 nm (**B**–**D**) generally cannot be seen for isolated metal oxide nanorods. Interestingly, for the Sb:SnO_*x*_ nonorod arrays, these resonances are observed both for s- (black) and p-polarized (blue) irradiance with slight deviations in intensity and peak position (Figure 3c).

To investigate the origin of these absorption peaks, full-wave simulations are performed in COMSOL Multiphysics (Figure 3d–f).^47^ The refractive index information for the core^48,49^ and shell^50^ are taken from literature data and further simulation parameters are given in Figure S9 (Supporting Information). To check for the effect of the core–shell structure, the optical response of the core and shell is first simulated independently (while the other material is treated as air). The simulated absorptance spectrum associated with the solely core nanorods (Figure 3d) resembles the important features of the measured spectrum for the core–shell structure, indicating that the optical properties of the metamaterial are mainly dominated by the nanorod cores. In turn, comparing the simulation of the array made from nanorod shells (Figure 3e) with the optical responses of the bare silicon substrate (Figure S10), it can be concluded that the nanorod shells have only a minor influence on the optical response. Simulations of the complete core–shell nanorods indicate that the absorption peaks observed for the core’s redshift are due to the presence of the nanorod shells. This effect can be explained by the high permittivity of the shells and therefore a change in the dielectric surrounding of the nanorod core.^51^ As observed from the experimental spectra, the simulations show three absorption peaks in the visible range (**B**–**D**) and one wide absorption peak in the NIR range (**A**). In comparison to the simulations, the peaks are slightly shifted. This shift can be explained by the nonuniformity of the real nanowire arrays. Additionally, the partly hollow structure (Figure 1d) can have a minor influence on the peak positions and intensities as confirmed via simulations (Figure S10).

To investigate the origin of the modes, the electric field distribution in the metamaterial at the wavelength of the absorption peaks (**A**–**D**) is evaluated (Figure 3g,h). For s-polarized illumination, the polarization is assumed along the *y*-axis, and the oscillation is in-plane concerning the metamaterial surface. For the absorption peaks B-D, the simulations indicate a strong field enhancement in the cavities between the nanorods with a coupling direction along the *y*-axis. Such cavity modes are typical for nanorod arrays with spacings below 50 nm.^24^ When the inter-rod gaps are sufficiently small, the transverse LSPRs of neighboring nanorods become highly coupled, and the metamaterial begins to behave similarly to a MIM waveguide.^25^ Modes that are laterally confined between neighboring nanorods form a series of longitudinal standing waves with an increasing number of harmonics at higher frequencies.^23^ As apparent from the side-view of the field distribution, the absorption peaks B–D represent the fifth, seventh, and ninth harmonic modes of the cavity resonances, respectively. Lower-order cavity resonance modes would be expected at higher wavelengths. Considering that the substrate is strongly influencing the resonance conditions,^23^ the change in optical properties of the silicon substrate above its band gap energy (1100 nm) can shift resonant modes in the spectrum governed by the metamaterial geometrical parameters. For example, a systematic variation of nanorods’ height can result in increasing the number of reflectance minima and generation of lower order resonances. The behavior of the A-mode significantly differs from that of the others. Excitation at the NIR peak wavelength results in a significant field enhancement within the nanorods, typical for the transversal LSPRs.

For p-polarized excitation, the optical electrical field is linearly polarized along the *x*-direction and, due to the oblique incidence, it also exhibits a component along the *z*-direction. Similar to the discussion for the s-polarization, the **B**–**D** modes can be ascribed to the fifth, seventh, and ninth harmonics of the cavity resonances. Whereby, the effective coupling direction is along the *x*-axis and split into two components (indicated by the red arrows), since the enhancement is strongest for the smallest gap spacings (Figure 3h). In summary, the simulations show that two different types of resonance modes are present in the nanorod metamaterial. In the visible range, cavity resonances are observed that are comparable to MIM waveguide modes, whereas the absorption peak in the NIR can be ascribed to the transversal LSPR of the nanorods.

As described previously, the plasmon frequencies in highly doped oxides can be tuned postsynthetically through fully reversible electrochemical doping, realizing electrical manipulation of SPR-based features.^34,36,37^ To actively modulate the plasmon resonances of the nanorods, they were charged with electrons in an electrochemical cell with a Li^+^-containing electrolyte and a platinum counter electrode (Figure 4a, Supporting Information). Applying a negative voltage to the array forces electrons to accumulate inside the nanorods, and Li^+^-ions accumulate in the gaps between the nanorods to ensure charge balance. The charging of the nanorods is monitored by cyclic voltammetry (Figure 4b). The fact that the cyclic voltammogram does not exhibit the typical rectangular shape of a pure double-layer capacitance^52^ indicates that intercalation processes of Li^+^ into the Sb:SnO_*x*_ nanorods are present (Figure 4a).^40^

**Figure 4:**
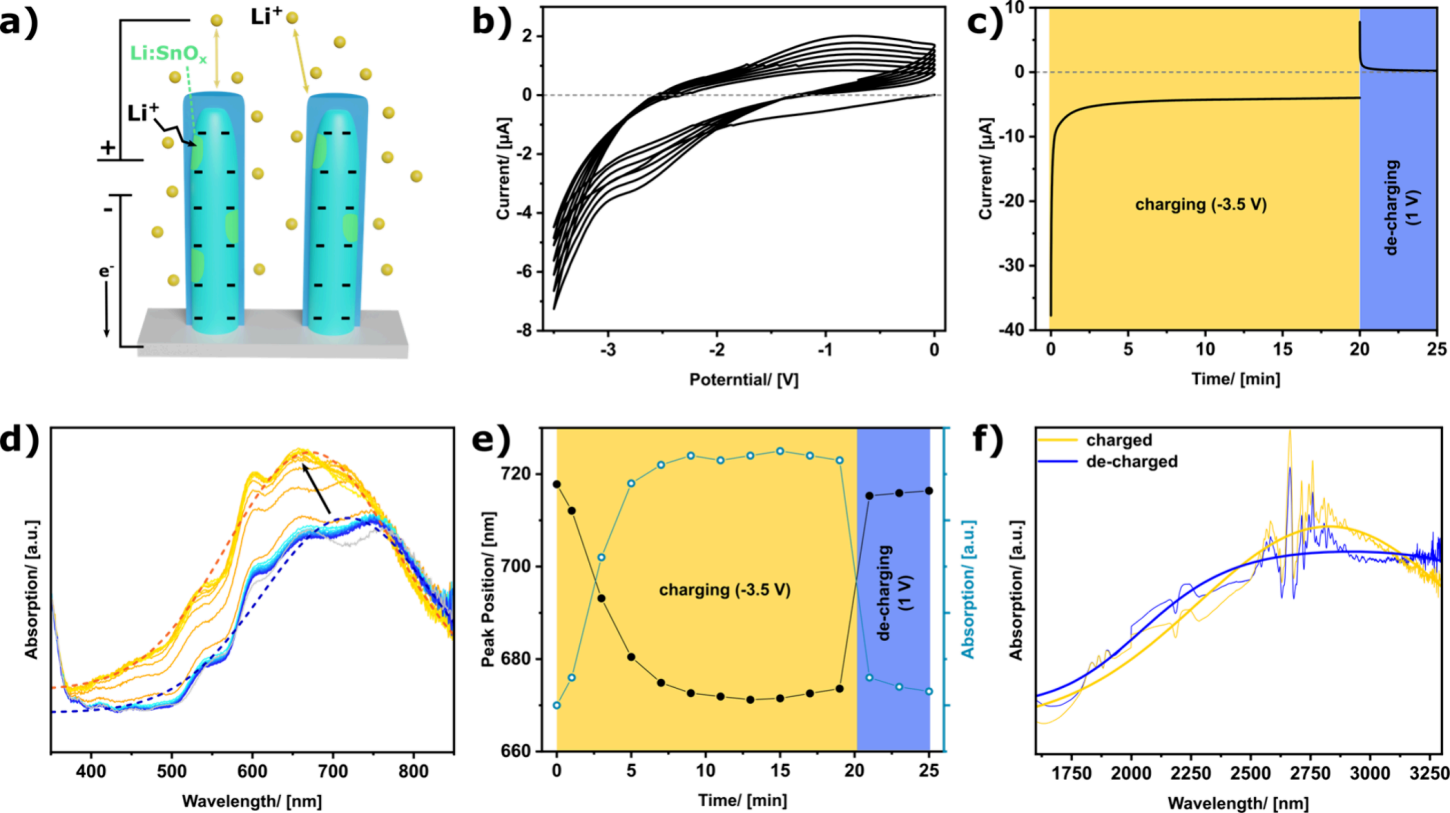
Schematic drawing of the charging process during the electro-optical modulation (a). Charge balancing by Li^+^ ions (yellow dots) and lithium intercalation (black arrow, green areas) are observed during the charging process. C–V measurement of the nanorod array in the Li^+^ containing electrolyte (b). Current flow with time during the charging and decharging process at a constant potential (c). UV–vis spectra measured with an interval of 2 min during the charging (orange to yellow) and decharging (light blue to dark blue) process (d). The dotted lines represent the peak fitting for the fully charged (orange) and decharged (blue) state. Peak position and absorption extracted from peak fitting during charging and decharging (e). Absorption spectra in the NIR region with Gaussian fit of the fully charged (yellow) and decharged (blue) state (f).

At the beginning of the charging process (application of −3.5 V), high currents are observed due to the capacitive charging of the electrochemical double-layer (Figure 4c, yellow area). Afterward, the current exponentially decreases and reaches a plateau typical of an intercalation process. During the intercalation process, the Li^+^-ions alloy with the metallic Sn present in the nanorods, forming a Li_*x*_Sn alloy. During decharging the current drops to nearly zero within a few seconds (Figure 4c, blue area), indicating that the slow electrochemical intercalation process is not reversible under these conditions and some Li remains in the nanorods, as also indicated by the cycle-dependent shape of the cyclic voltammogram of the decharging process (Figure S11). The presence of Li in the nanorods after the decharging process is confirmed by XPS, and a ratio of 0.087 Li/Sn is observed (Figure S12). Since the optical spectrum after the discharging process resembles the pristine spectrum, this residual Li seems to have minor effect on the optical properties of the metamaterial (Figure 4d). Additionally, no significant reduction and lithiation of the tungsten oxide underlayer is observed, ruling out an electrochromic alteration of the tungsten oxide (Figure S13). For future studies, it may be beneficial to utilize a lithium-free electrolyte to rule out a disturbance by Li intercalation fully.^36^ Immersion of the nanorod array in the electrolyte leads to an overlap and shift of the absorption peaks observed in the visible range (Figure 4d, gray). When the nanorod arrays are submerged in the propylene carbonate electrolyte, the cavities between the nanorods get filled with the electrolyte, and due to the higher refractive index of propylene carbonate (*n* = 1.4189) compared to air (*n* ≈ 1), the resonance conditions for the cavities change.^23^ Due to their broadening, several cavity modes seem to overlap in the observed wavelength range, forming a combined peak with a fringy shape (Figure 4d). Application of a negative bias (−3.5 V) results in a blue shift and an increase of the absorption peak with time (Figure 4d, orange to yellow). The plasmonic modulation is not prompt, but it takes around 8 min until a constant state is reached. Subsequent decharging of the nanorods by a positive bias (1 V) is accomplished within 2 min, and the spectrum after the decharging resembles the pristine one (Figure 4d, bright blue to blue), indicating a fully reversible modulation of the cavity modes. UV–vis evaluation of a consecutive charging and discharging cycle confirms the fully reversible manner of the optical switching (Figure S14). The absorption peaks are fitted with a Gaussian profile (Figure 4d, dotted lines). In the pristine state, the maximum of the absorption peak is observed at 718 nm (Figure 4e). During the charging, the maximum blue-shifts to 672 nm, accompanied by an increase of 22% in maximum absorption (Figure 4e). The values extracted from the peak fitting for each time step are summarized in Table S1. Both changes are consistent with a modulation of the free carrier concentration in the nanorod cores and an increase in their plasma frequency, due to carrier accumulation. Compared to the nanorod array in air (Figure 3b), the peak position of the transversal plasmonic resonance is also slightly shifted and the peak is broadened, due to the increase in the refractive index of the environment. During charging, the intensity of the peak in the NIR also increases by about 26% (Figure 4f). This effect can be explained by an increase in charge carrier density in the nanorods.^35,40^ So, the core–shell structure in combination with the arrangement of the nanorods, not only introduce cavity resonance modes in the nanostructured material, but these coupled resonances are additionally electrochemically addressable. This enables fully reversible electro-optical modulation of cavity resonance modes of the metamaterial, and in the future, for electrochemical charging-induced plasmons in undoped semiconductors.

In summary, by introducing a controlled thermal oxidation process, we fabricated nanorod arrays featuring a localized surface plasmon resonance (LSPR) in the NIR range. Characterization of the nanorods reveals a core–shell structure with a highly doped Sb:SnO_*x*_ (*x* ≈ 1) core and a Sb:SnO_*x*_ (*x* ≈ 2) shell. Optical full-wave simulations and experimental absorption spectra show that the metamaterial exhibits cavity resonance modes, based on the coupling of transversal plasmon resonances of adjacent nanorods. The cavity modes and the LSPR can be dynamically tuned by electrochemical charging of the nanorod arrays, which increases the charge carrier concentration in the nanorod cores. Tuning the number and position of such cavity modes can find application in nonlinear and switchable devices based on the spatial-dependent control of electric field hot-spots inside the metamaterial. This process of electron accumulation in the nanorods is reversible, paving the way to electro-optical modulation of anisotropic multi-mode plasmonic metamaterials.

## Supplementary Material



## Notes

Additional experimental details, materials, and characterization methods, including further nanostructure characterization, electrical investigation, and optical simulation data (PDF)
