# Supplementary material for: Dynamic Modulation of Coupled Plasmon Resonances in Antimony-Doped Tin Oxide Nanorod Metamaterial by Charge Carrier Injection
Source: Nano Lett. 2025 May 19;25(21):8628–35. doi: 10.1021/acs.nanolett.5c01485 (PMC12123670; doi:10.1021/acs.nanolett.5c01485)
Supplement: Supplementary file 1 [file nl5c01485_si_001.pdf]

Dynamic Modulation of Coupled Plasmon Resonances in Antimony-doped Tin Oxide Nanorod Arrays by Charge Carrier Injection

Thomas Herzog,[a,b] *Atefeh Habibpourmoghadam,*[b,c,d*] *Nele Pannewitz,*[a] *Yaşar Krysiak,*[a] *Irene Morales,*[a,b] *Sonja Locmelis,*[a] *Antonio Calà Lesina,*[b,c,d] *Sebastian Polarz*[a,b*]

[a]Institute of Inorganic Chemistry, Leibniz University Hannover, Callinstraße 3-9, 30167 Hannover, Germany

[b]Cluster of Excellence PhoenixD, Leibniz University Hannover, Welfengarten 1A, 30167 Hannover, Germany

[c]Hannover Centre for Optical Technologies, Leibniz University Hannover, Hannover, 30167, Germany

[d]Institute for Transport and Automation Technology, Leibniz University Hannover, Garbsen, 30823, Germany

[*]E-mail: sebastian.polarz@aca.uni-hannover.de

[*]E-mail: atefeh.habibpoor@hot.uni-hannover.de

SI Text 1. Material and Methods

Template Preparation

The anodic aluminum oxide (AAO) templates are grown directly on a silicon wafer. Therefore, the wafer is coated with an 8 nm Ti adhesive layer, a 50 nm tungsten interlayer and a 1000 nm Al layer by magnetron sputtering. The wafer is sliced into 1.4 x 1.4 cm pieces to fit in the homemade sample holder for the electrochemical anodization process. The two-step anodization is carried out in 0.3 M oxalic acid at a constant voltage of 40 V. The temperature of the electrolyte is kept constant at 5 ˚C during the whole anodization process. The first anodization is carried out for 5 min and subsequently the distorted AAO is removed in a 0.3 M CrO_3/H_3PO_4 bath at 60 ˚C for 45 min. The second anodization is carried out under the same conditions as the first until a sharp decrease in current is observed, due to the full conversion of Al to Al_2O_3 and the starting oxidation of the tungsten interlayer. To widen the pores and to remove the barrier oxide (Al_2O_3 and WO_3) the templates are immersed in 5%wt H_3PO_4 for 25 min and in phosphate buffer solution (pH = 7) for 10 min.

Electrodeposition of Metallic Nanorods

For the electrodeposition of the metallic nanorods the templates are immersed in the corresponding electrolyte in the homemade sample holder and an Ag/AgCl reference electrode is immersed in the electrolyte. For the deposition of pure tin nanorods a solution of 70 mM sodium citrate tribasic dihydrate, 100 mM citric acid monohydrate and 15 mM $SnSO_4$ in degassed and deionized water is used. For the deposition of the tin-antimony alloy nanowires a second solution of 70 mM sodium

citrate tribasic dihydrate, 100 mM citric acid monohydrate and 15 mM $Sb_2(SO_4)_3$ in degassed and deionized water is prepared and 1 mL of the Sb-solution is mixed with 19 mL of the Sn-solution to achieve a doping ratio of 10% Sb in Sn. All solutions are prepared directly before the electrodeposition process. The electrodeposition of the nanorods is carried out in a pulsed manner in galvanostatic mode for both the pure tin and the tin-antimony alloy nanorods. For the deposition a current of -4 mA is forced between the working and counter electrode for 0.1 s, followed by a standby pulse of 0 mA for 0.1 s to allow diffusion of the metal ions into the pores of the AAO template. To reach the desired nanorod length 1500 deposition cycles are conducted. After the deposition the templates are removed from the sample holder and rinsed with deionized water.

Oxidation of Fully Confined Nanorods (Approach 1)

For approach 1 the nanorods in the template are thermally oxidized directly after the electrodeposition without further modification. The oxidation is conducted in air in a three-step process. First the sample is heated to 200 ˚C within 1 h and kept at this temperature for 3 h to grow a thin SnO layer at the nanorod surface. Afterwards the nanowires are heated to 400 ˚C within 1 h and kept at this temperature for 3 h. In the last step a temperature of 600 ˚C is reached within 1 h with a hold time of 12 h. After that the samples are allowed to cool down naturally and are removed from the muffle furnace, when the temperature drops below 40 ˚C.

Oxidation of the Nanorods with Oxygen Diffusion Path (Approach 2)

Oxygen diffusion path next to the nanorods are grown by immersing the samples in a solution of 0.33 M CrO_3 and 0.03 M HCl in deionized water for 15 min. Thereby the aluminum oxide around the nanorods is slightly etched and voids next to the nanorods are generated without fully removing

the template (Figure S2). Additionally, a thin SnO_x layer is grown on the nanorod surface. After careful rinsing with deionized water the samples are thermally oxidized in the same way as for approach 1.

Removal of the Template for Electrochemical Charging Experiments

After the oxidation the template is removed by immersion in solution of 0.33 M CrO_3 and 0.03 M HCl in deionized water for 1 h to fully remove the template and get the free-standing nanorod arrays, that are used for the optical and electrochemical measurements.

Characterization Methods

An ATC Orion 5 UHV sputter system was employed for sputter coating. SEM images and EDX measurements were acquired using a Hitachi Regulus SU8230 equipped with an Oxford Ultim Max 100 EDX detector. UV-Vis-NIR measurements were carried out with a Cary 5000 spectrometer (Agilent Technologies Inc.) equipped with a praying mantis setup for measurement in diffusive reflectance with the incidence beam at an oblique incidence of 45°. The spectra are referenced to a highly-reflective mirror-finished aluminum thin film, enabling direct comparison of the intensities of the features of both samples. The extinction measurements are baseline-corrected with an aluminum mirror as reference. XPS spectra were obtained with a PHI 5000 VersaProbe III X-ray photoelectron spectrometer and sputtering was conducted with an Ar ion gun with an acceleration voltage of 3 kV. XRD measurements were conducted with a Bruker D8 Advance. Polarization dependent reflection/extinction measurements were obtained with a PerkinElmer Lambda1050 spectrometer equipped with an URA accessory. Electrochemical

measurements and the electrodeposition was conducted either with a Keithley 2450 SourceMeter or an Methrom Autolab Potentiostat/Galvanostat PGSTAT10.

Electrochemical and Electro-Optical Measurements

For the electrochemical measurements the nanorods are contacted through the underlaying tungsten layer and immersed in a 0.1 M Lithiumbis(trifluormethylsulfonyl)amid (LiTFSI) in propylene carbonate electrolyte (**Scheme M1**). The potential is applied between the sample and a platinum counter electrode. For electro-optical measurements the whole setup is installed in the UV-Vis spectrometer in a homemade electrolytic bath and the sample is immersed in a thin film of the electrolyte. The measurements are conducted in reflectance geometry with a praying mantis setup for measurements in diffusive reflectance. The potential is applied by a Keithley 2450 SourceMeter in a two-electron configuration.

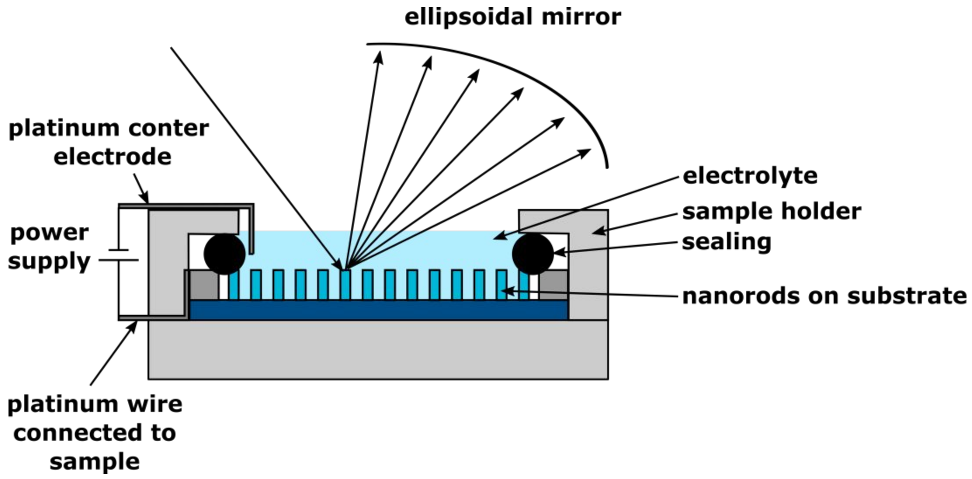

Scheme M1. Schematic drawing of the setup for the electro-optical measurements.

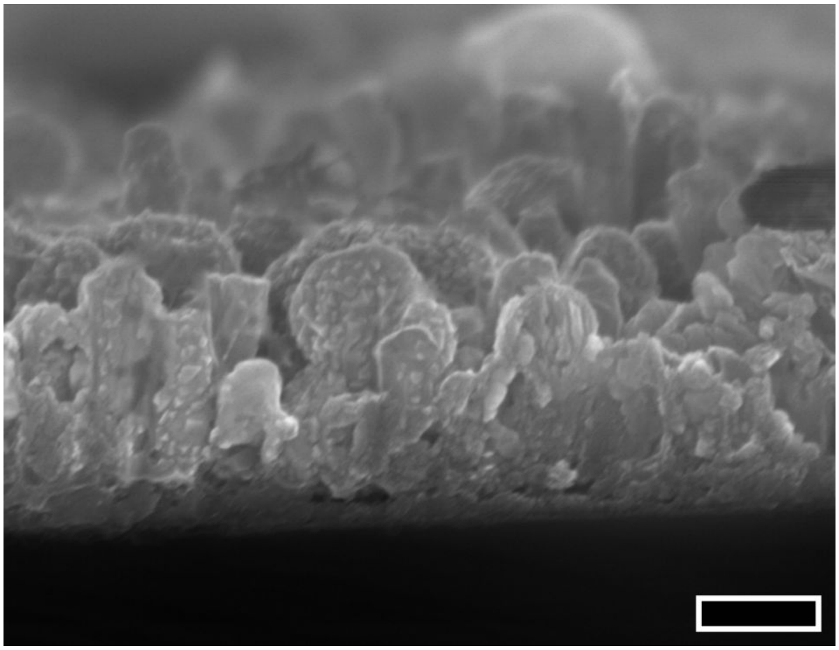

Figure S1. SEM image of nanowire array after thermal oxidation in air without template (scale bar 200 nm).

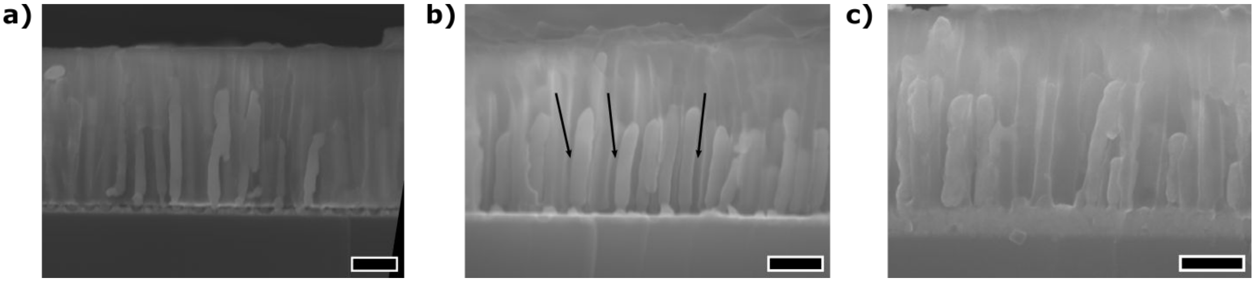

Figure S2. Three states of the oxidation process according to approach 2 (scale bars 200 nm). The as-prepared metal alloy nanowires before the partly etching of the template (a). Side-view SEM image of the alloy nanowires after partly etching of the template (b), the black arrows indicate the diffusion channels for oxygen, that are generated during the etching. After thermal oxidation no voids are visible in the lower part of the oxidized nanorods (c).

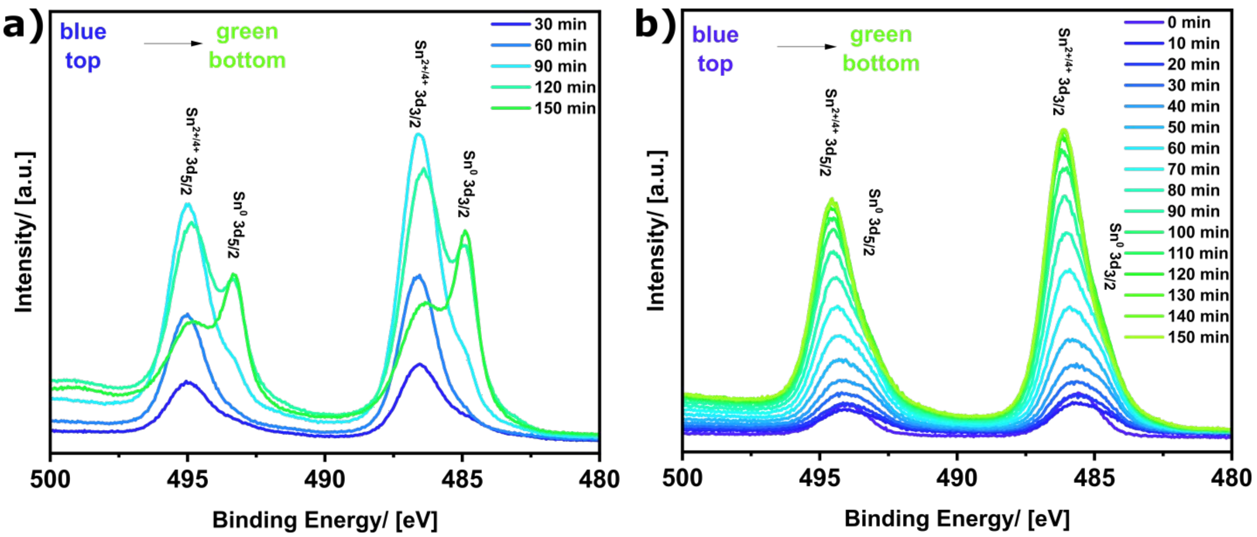

Figure S3. Sn 3d core level spectra of the oxidized nanorods for approach 1 (a). Spectra are recorded before and after Ar-ion sputtering for 30 min intervals to reveal oxidation gradient from top to bottom of the nanorods. Sn 3d core level spectra of the oxidized nanorods for approach 2 (b). Spectra are recorded before and after Ar-ion sputtering for 10 min intervals to reveal oxidation gradient from top to bottom of the nanorods.

The XPS spectra are recorded with the nanorods still in the template and argon ion sputtering was applied to successively remove the top layer of the template- nanorod composite film, to analyze the upright standing nanorods from their top to the bottom. Since the nanorods do not reach the top of the template the sputtering time was set to 0 min for the first Sn spectrum, which indicates a noticeable amount of Sn. The sputtering was conducted until no Sn signal was detected anymore, indicating that the whole SnO_x nanorods and the template was removed by sputtering. To visualize the oxidation gradient in Figure 1f the $Sn3d_{3/2}$ peaks of the spectra from Figure S3 are fitted with a Gaussian peak profile and a Shirley baseline. For both approaches the peak is deconvoluted in

SnO_x (at higher binding energies) and a Sn^0 peak (at lower binding energies). Form the fitting results the atomic percentage of SnO_x and Sn^0 is calculated and presented in Figure 1f.

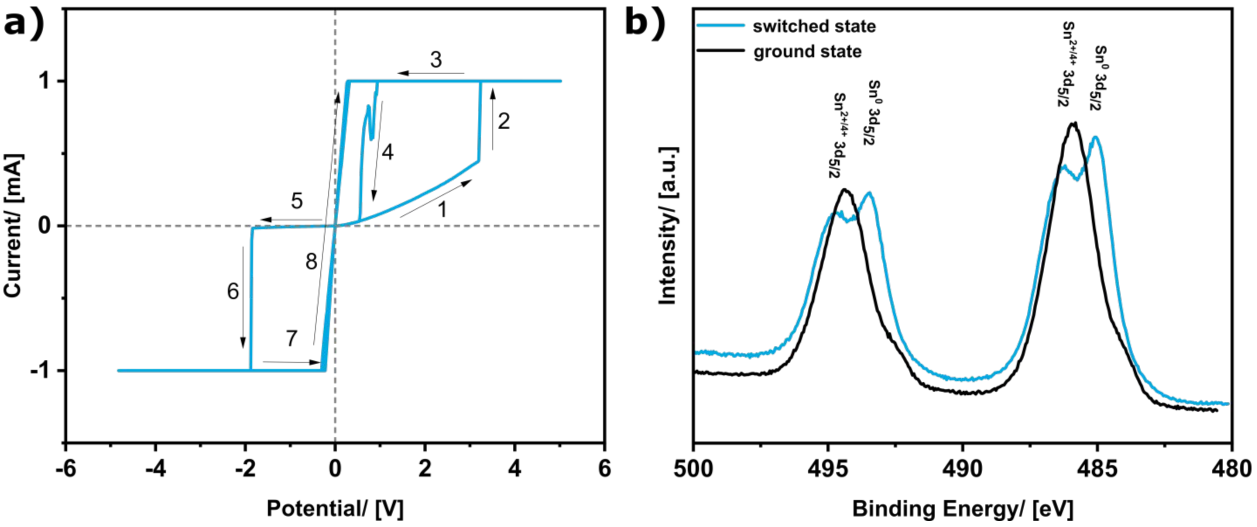

Figure S4. Current-Voltage curve of the nanorods prepared by approach 1 (a). The nanowire tops are contacted with silver conductive paste and the bottoms are contacted through the substrate. XPS Sn 3d core level spectrum recorded at the same depth for the sample in the pristine (black) and switched (blue) state (b).

If the nanowires from approach 1 are contacted by silver paste and through the substrate current-voltage profiles of the arrays can be measured. The current-voltage profile in Figure S4a shows the typical hysteresis for resistive switching processes.[1] The measurement course of the measurement is indicated by the arrows and numbers and a current compliance of 1 mA is applied for the measurements. During the first cycles several resistive switching events are indicated by sharp increase/decrease of the current, but after some cycles the system stays in the switched state (low resistance state) and no back-switching takes place anymore. The XPS Sn 3d core level

spectra in Figure S4b indicate that the switching process can be assigned to a reduction of SnO_x to Sn^0, when the nanorods are goring from the high resistance state to the low resistance state and vice versa. For this switching process an oxygen gradient along the nanorod long axis is crucial.[1]

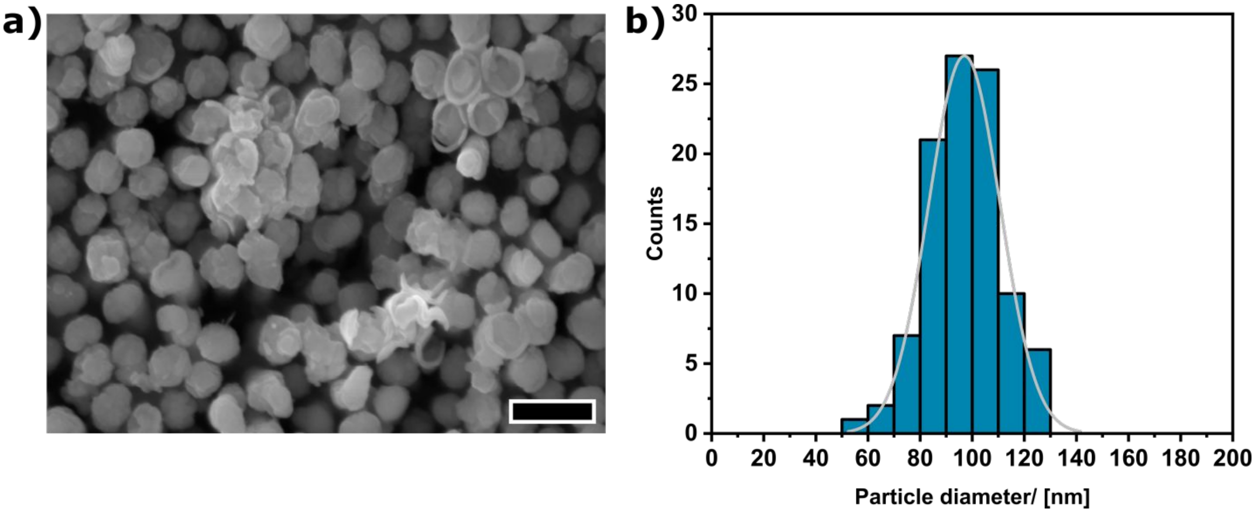

Figure S5. Top-view SEM image of the nanorods oxidized according to approach 2 after removal of the template (a, scalebar 200 nm). Nanorod diameter distribution extracted from (a), showing a mean diameter of 97 nm and a diameter range from 55 nm to 125 nm (b).

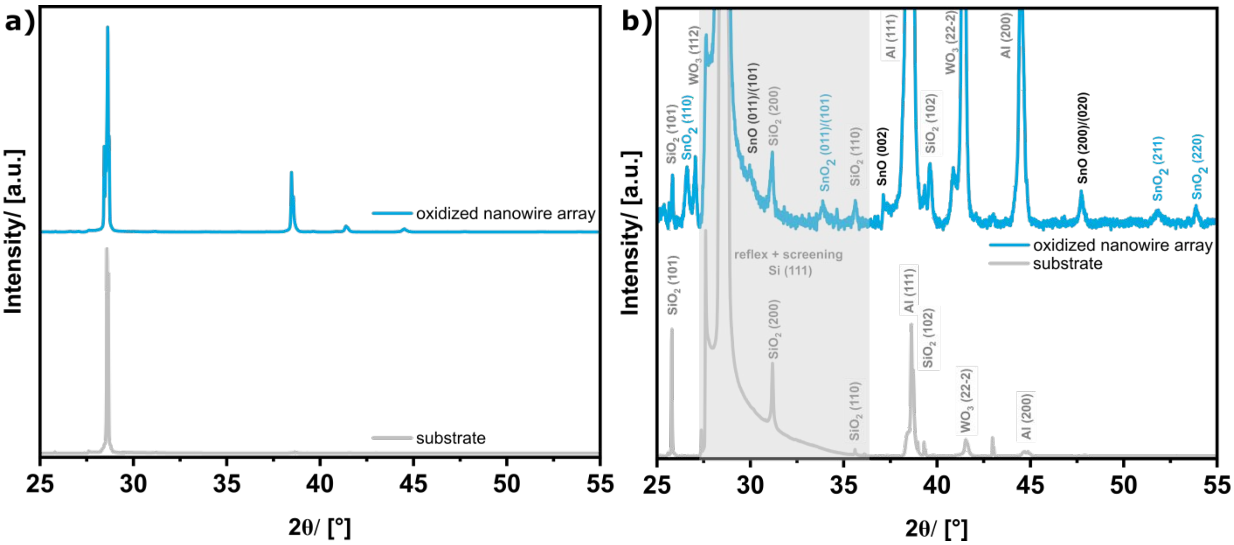

Figure S6. Full range XRD pattern (a) of the nanorod array (blue) and the tungsten and partly aluminum coated substrate (grey). Zoomed-in XRD pattern of the two samples (b) with indexing of the reflexes stemming from the substrate (Si, SiO_2, WO_3 and Al) and the reflexes from the oxidized nanorod array (SnO, SnO_2).

Electrodeposition of Sn in an AAO template results in a preferred growth in [100] direction, which can be derived from the larger intensity of the (200) reflex compared to the (101) reflex.[2] During the oxidation process the Sn gets partly melted and therefore the preferential orientation is expected to vanish, but the absence of the SnO (110) reflex indicates that there is still some orientation in the SnO domains.

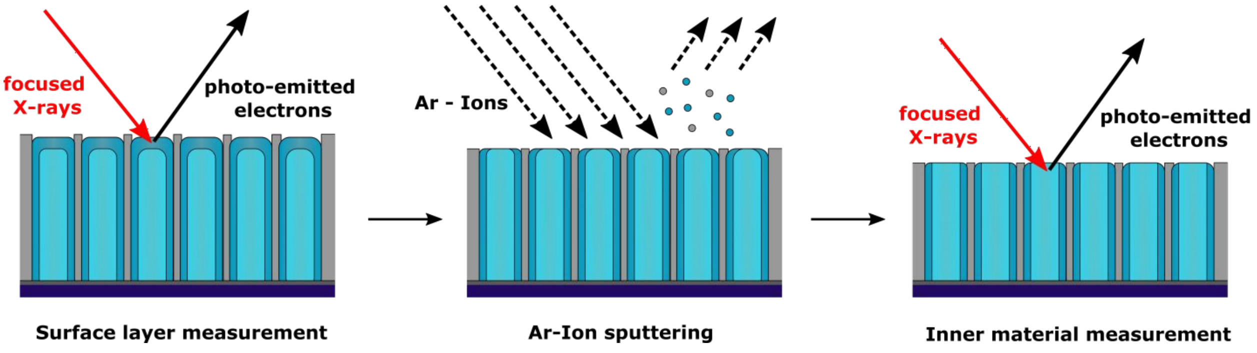

Figure S7. Schematic drawing of the XPS measurement of the surface layer and inner material of the nanorods with the Ar-ion sputtering process between the measurements.

The XPS measurements were carried out after the thermal oxidation process and before the AAO template was etched away. As described in the manuscript, during the thermal oxidation according to approach 2 the outer shell of the nanowires is oxidized, which results in the observed core-shell

structure. Thereby not only the sides of the nanorods, but also the top/cap of the nanorods are oxidized and are composed of the shell material. Since this shell is thicker than the penetration depth of the XPS measurement (~2 nm) the shell composition can be determined by carrying out the measurements directly after the thermal oxidation. For measurement of the interior of the nanorods the top/caps of the nanorods and the AAO template is removed by Ar sputtering, therefore the X-rays of the XPS directly reach the interior of the nanorods and its composition can be determined by analyzing the photo-emitted electrons. There may be also a contribution of the shell at the nanorod sides to the XPS signal, but as no peak for Sn^{4+} is observed in the spectrum the contribution of the shell is expected to be negligible.

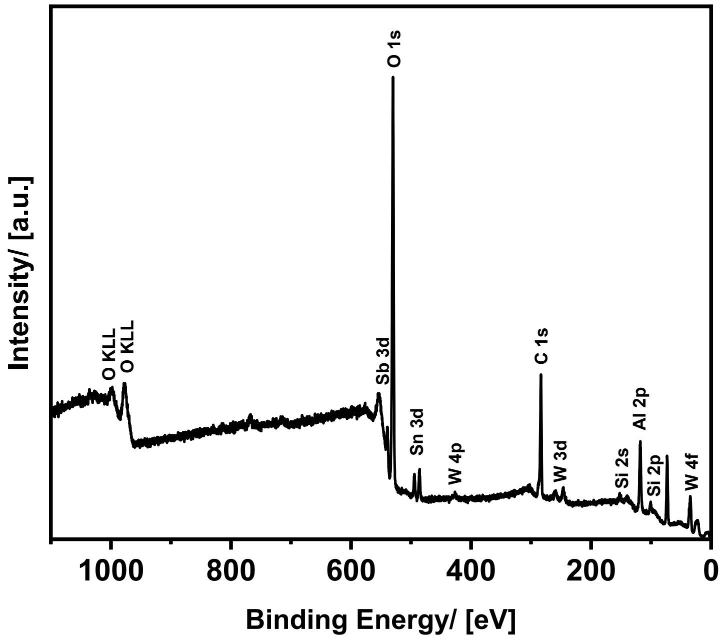

Figure S8. Survey XPS spectrum of the nanorod array after oxidation, indicating that no impurities are present and only signals stemming from the nanorods (Sn, Sb, O) and the substrate (Si, W, Al) are present.

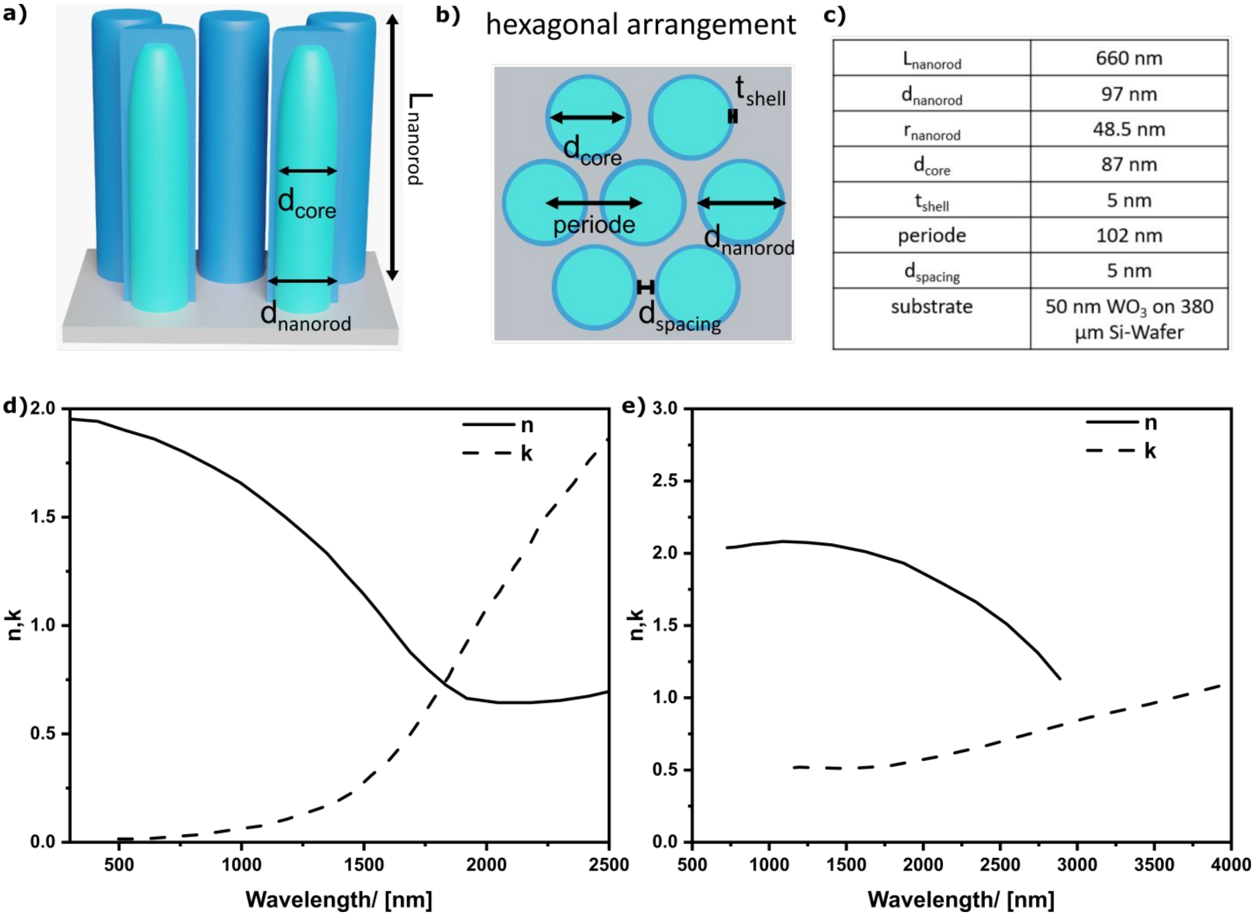

$L_{nanorod}$	660 nm
$d_{nanorod}$	97 nm
$r_{nanorod}$	48.5 nm
d_{core}	87 nm
t_{shell}	5 nm
periode	102 nm
$d_{spacing}$	5 nm
substrate	50 nm WO_3 on 380 µm Si-Wafer

Figure S9. Side-view (a) and top-view (b) schematic drawing of the structure used for optical simulations including geometrical simulation parameters (c). Refractive index (n) and absorption coefficient (k) of the core material[3,4] (a) and the shell material[5] (b) used for the optical simulations.

According to the measurement setup, the incident beam is assumed at oblique incidence of 45°. For the nanorods, an average core diameter of 87 nm, a shell thickness of 5 nm, and a length of 660 nm is assumed. The nanorods are arranged in a hexagonal array in an air environment on a tungsten oxide (50 nm) coated silicon wafer with a spacing of 5 nm.

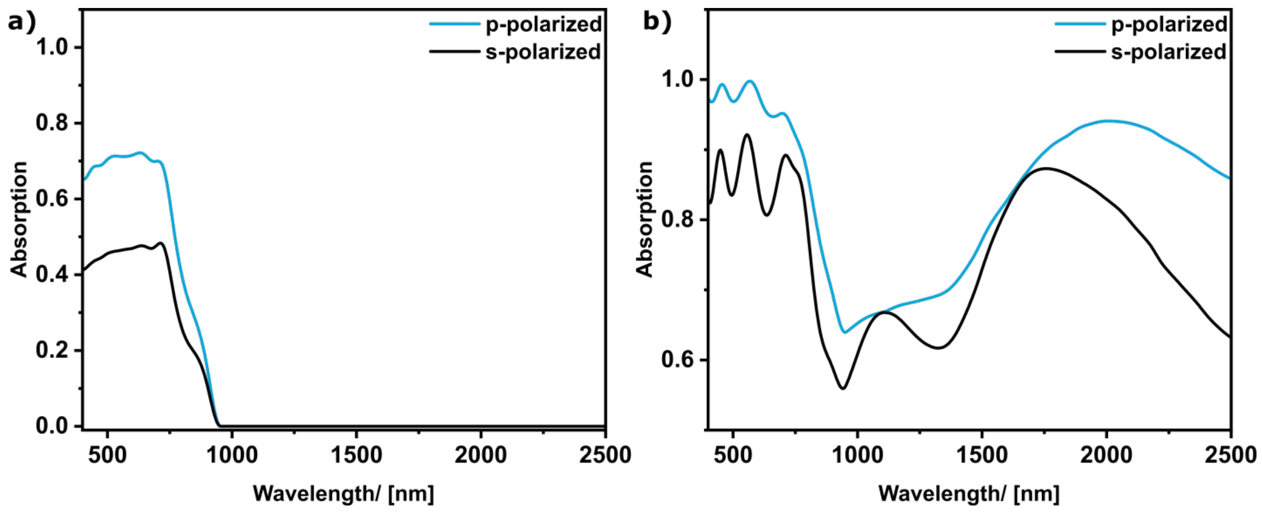

Figure S10. Simulated absorption spectrum for the silicon substrate without nanorods (a) and for the complete metamaterial assuming that the nanorods exhibit hollow cores (b) as indicted in Figure 1d for s- (black) and p-polarized (blue) irradiance, respectively.

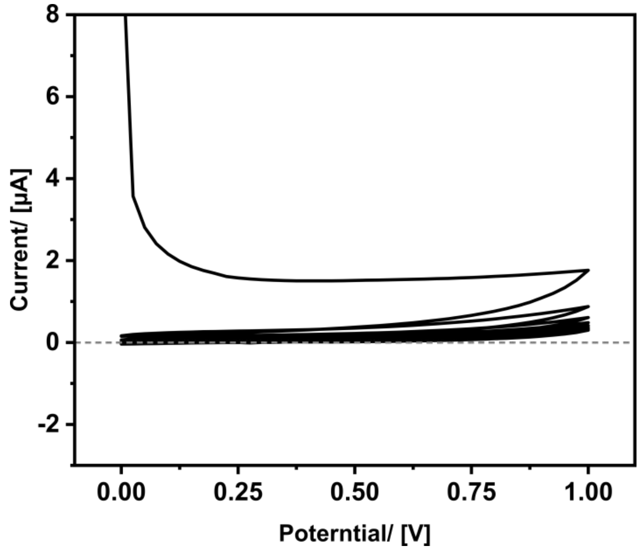

Figure S11. C-V measurement of the nanorod array in the Li^+ containing electrolyte in the positive voltage direction (de-charging process).

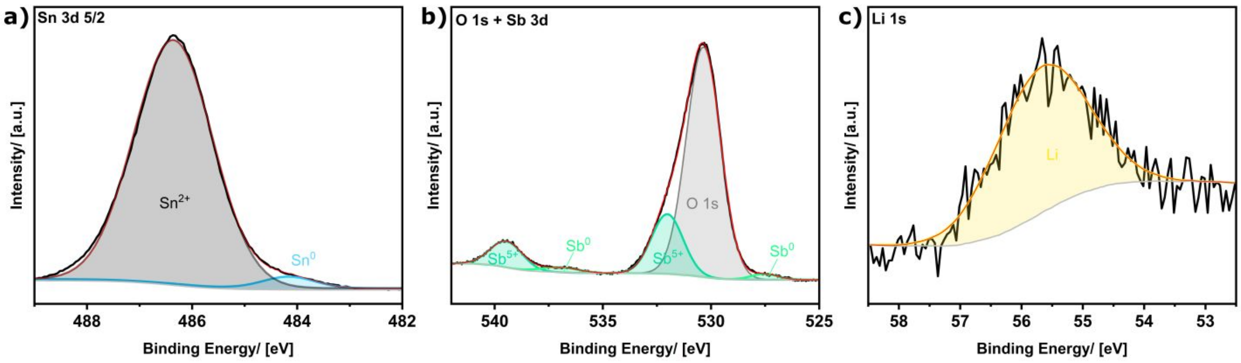

Figure S12. XPS Sn 3d 5/2 (a), O 1s + Sb 3d (b) and Li 1s (c) core level spectra of the nanorod array after de-charging process.

The contaminated top layer and the Sb:SnO_2 shell is removed by Ar-ion sputtering before the XPS spectra of the nanorod array are taken. The peaks are deconvoluted to identify the species present in the metamaterial. The Sn, Sb and O spectra show no significant differences compared to the ones o the pristine sample (**Figure 2**). In the core level spectrum of Li 1s the amount of residual intercalated Li after the de-charging process is observed and a ratio of 0.0869 Li/Sn is determined, indicating that the Li intercalation process is not fully reversible and a small amount of Li remains in the nanorods after the de-charging process.

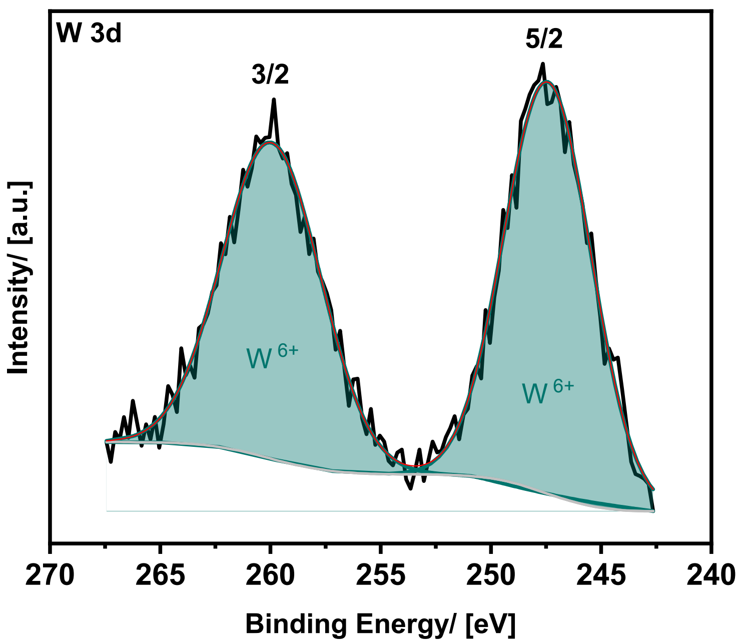

Figure S13. XPS W 3d core level spectrum of the metamaterial after charging process.

The W $3d_{5/2}$ and the W $3d_{3/2}$ peak can be fitted by a single gaussian fit, indicating only one tungsten species present in the material. The binding energies of the W $3d_{5/2}$ and the W $3d_{3/2}$ are determined to be 247.5 eV and 259.9 eV, respectively, resulting in a separation of 12.4 eV. These binding energies and the peak separation are perfectly fitting to WO_3, indicating that the WO_3 layer is not getting reduced during the charging process and no significant lithium intercalation into the WO_3 is expected.[6]

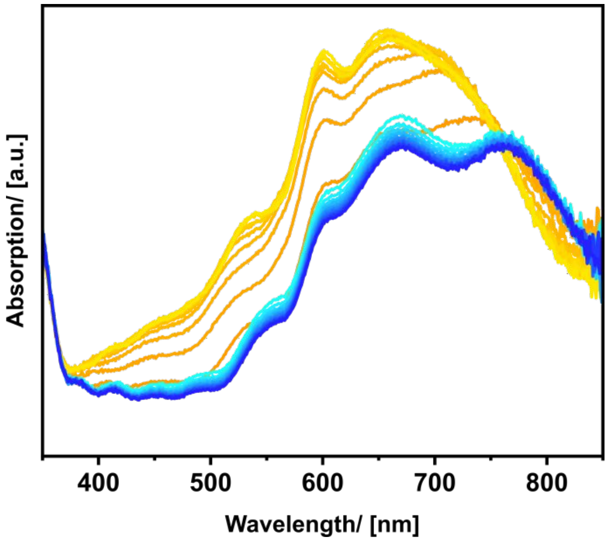

Figure S14. UV-Vis spectra measured with an interval of 2 min during a consecutive charging (orange to yellow) and de-charging (light blue to dark blue) cycle, indicating the fully reversible optical switching.

Table S1. Peak position, absorption maximum and full width at half maximum (FWHM) values extracted by gaussian fitting of the absorption peak during the electrochemical charging and de-charging process. The absorption maximum is measured in reference to a mirror finished aluminum reference.

Time [min]	Peak position [nm]	Absorption [a.u.]	FWHM [nm]
0	717.81	2.4	250.1
1	712.08	2.46	251.3
3	693.13	2.72	250.6
5	680.44	2.88	245.5
7	674.86	2.92	243.4
9	672.63	2.94	244
11	671.9	2.93	244.8
13	671.2	2.94	243.9
15	671.5	2.95	242.4
17	672.57	2.94	243.4
19	673.61	2.93	246.1
21	715.31	2.46	251
23	715.9	2.44	250.5
25	716.4	2.43	251.4

REFERENCES

(1) Herzog, T.; Weitzel, N.; Polarz, S. Oxygen Vacancy Injection-Induced Resistive Switching in Combined Mobile and Static Gradient Doped Tin Oxide Nanorods. *Nanoscale* **2020**, *12* (35), 18322–18332. https://doi.org/10.1039/D0NR03734F.

(2) Luo, B.; Yang, D.; Liang, M.; Zhi, L. Large-Scale Fabrication of Single Crystalline Tin Nanowire Arrays. *Nanoscale* **2010**, *2* (9), 1661–1664. https://doi.org/10.1039/C0NR00206B.

(3) Mwamburi, M.; Wäckelgård, E.; Roos, A. Preparation and Characterisation of Solar Selective SnOx:F Coated Aluminium Reflector Surfaces. *Thin Solid Films* **2000**, *374* (1), 1–9. https://doi.org/10.1016/S0040-6090(00)01045-2.

(4) Stjerna, B. A.; Granqvist, C.-G. Optical and Electrical Properties of Doped Rf-Sputtered SnOx Films. In *Optical Materials Technology for Energy Efficiency and Solar Energy Conversion XI: Selective Materials, Concentrators and Reflectors, Transparent Insulation and Superwindows*; SPIE, 1992; Vol. 1727, pp 178–193. https://doi.org/10.1117/12.130505.

(5) Shanthi, S.; Subramanian, C.; Ramasamy, P. Investigations on the Optical Properties of Undoped, Fluorine Doped and Antimony Doped Tin Oxide Films. *Crystal Research and Technology* **1999**, *34* (8), 1037–1046. https://doi.org/10.1002/(SICI)1521-4079(199909)34:8<1037::AID-CRAT1037>3.0.CO;2-J.

(6) Sarma, D. D.; Rao, C. N. R. XPES Studies of Oxides of Second- and Third-Row Transition Metals Including Rare Earths. *Journal of Electron Spectroscopy and Related Phenomena* **1980**, *20* (1), 25–45. https://doi.org/10.1016/0368-2048(80)85003-1.
